# The etiology of cardiac hypertrophy in infants

**DOI:** 10.1038/s41598-021-90128-3

**Published:** 2021-05-19

**Authors:** Raymond Stegeman, Nina D. Paauw, Rosalie de Graaf, Rosa L. E. van Loon, Jacqueline U. M. Termote, Johannes M. P. J. Breur

**Affiliations:** 1 Department of Pediatric Cardiology, Wilhelmina Children’s Hospital, University Medical Center Utrecht, PO BOX 85090, 3584 EA Utrecht, The Netherlands; 2 Department of Genetics, University Medical Center Utrecht, Utrecht, The Netherlands; 3 Department of Neonatology, Wilhelmina Children’s Hospital, University Medical Center Utrecht, PO BOX 85090, 3584 EA Utrecht, The Netherlands

**Keywords:** Cardiovascular diseases, Paediatric research, Cardiology, Medical research

## Abstract

This study aimed to describe the variety of etiologies currently identified in infants with cardiac hypertrophy (CH) and investigate whether there is a relation with hyperinsulinism, echocardiographic characteristics and prognosis. This retrospective cohort study included infants born between 2005 and 2018 with CH measured by echocardiography [interventricular septum (IVS) and/or left ventricular posterior wall (LVPW) thickness with *Z*-score ≥ 2.0]. Children with congenital heart disease or hypertension were excluded. Underlying diagnosis, echocardiographic and follow-up data were extracted from patient files. Seventy-one infants with CH were included. An underlying cause of CH was identified in two-thirds (n = 47). Most common etiologies of CH were malformation syndromes (n = 23, including Noonan n = 12) and maternal diabetes mellitus (n = 13). Less common causes were congenital hyperinsulinism (n = 3), metabolic- (n = 5), sarcomeric- (n = 2) and neuromuscular disease (n = 1). In half of the identified causes (n = 22) an association with hyperinsulinism was described, including maternal diabetes mellitus (n = 13), malformation syndromes with insulin resistance (n = 6) and congenital hyperinsulinism (n = 3). CH associated with hyperinsulinism was echocardiographically characterized by lower LVPW thickness, higher IVS:LVPW ratio and more frequent sole involvement of the IVS (all, *p* ≤ 0.02). CH associated with hyperinsulinism normalized more often (41 vs. 0%) with lower mortality rates (14 vs. 44%) compared to CH not associated with hyperinsulinism (all, *p* ≤ 0.03). Nowadays, an etiology of CH can be identified in the majority of infants. The development of CH is often associated with hyperinsulinism which is mainly characterized by focal hypertrophy of the IVS on echocardiography. Prognosis depends on the underlying cause and is more favorable in CH associated with hyperinsulinism.

## Introduction

In infants cardiac hypertrophy (CH) is a rare finding on echocardiographic examination. It can be either a sign of enlarged cardiomyocytes or of hypertrophic cardiomyopathy (HCM) in which there is histological and functional disruption of the myocardial structure/composition in the absence of abnormal loading conditions, as congenital heart disease or hypertension^1,2^. In previous literature CH is often incorrectly described as HCM which might be explained by the fact that CH cannot be distinghuished completely from HCM using echocardiographic examination. Therefore in the remainder of this article both CH and HCM will be reffered as CH^3^.

CH in infants has an incidence of 30 per million and is diagnosed with echocardiography requiring a left ventricular wall thickness of more than 2 standard deviations greater than the predicted mean corrected for body surface area (Z-score ≥ 2.0)^4–8^. The degree and distribution of CH vary between the different etiologies and include diastolic interventricular septal (IVS) hypertrophy and/or left ventricular posterior wall (LVPW) hypertrophy with or without left ventricular outflow tract obstruction (LVOTO)^9^. The prognosis is variable and depends on the underlying disorder. Children with CH diagnosed before 1 year of age seem to have higher mortality rates than those diagnosed after 1 year of age^10–12^.

The etiology of CH in young childhood is diverse and the mechanisms of development and the distribution of etiologies in young children are supposedly different from CH developing later in childhood^11,13^. Known underlying causes of CH in infants include malformation syndromes (like Noonan), metabolic diseases (mitochondrial disorders and storage diseases such as Pompe), sarcomeric diseases (mutations in cardiac sarcomeric contractile protein genes MYH7, MYBPC3) and neuromuscular disorders (such as Friedreich ataxia). In our recent literature review we already emphasized the importance to additionally consider hyperinsulinism in the differential diagnosis as hyperinsulinism is widely associated with CH. We report an occurrence of CH in 13–44% of infants of diabetic mothers, in approximately 40% with congenital hyperinsulinism and in insulin resistence syndromes^14^.

Unfortunately, literature reports that in a large group of young children the etiology of CH remains unknown, while determining the right diagnosis is important since this has direct implications for prognosis and possible medical treatment^8,11–13,15,16^. Therefore, the main objective was to describe the current spectrum of identified etiologies of CH in our population of infants over the past years. In addition we aimed to investigate whether echocardiographic characteristics might help to distinghuish between underlying causes and relate to the prognosis of CH in infants.

## Material and methods

### Study design and population

This is a tertiary single center retrospective study performed in Wilhelmina Children’s Hospital, Utrecht, The Netherlands. Echocardiographic reports of all consecutive infants (≤ 1 year) born between 2005 and 2018 and at least 1 year of follow-up were screened for the presence of CH. Their echocardiograms were reassessed to determine whether they met the criteria of CH. Children with CH as a result of hypertension or underlying congenital heart disease were excluded. Data of infants with CH were extracted from patient files. The study was performed in accordance with relevant guidelines and regulations. Study quality was checked before submission to the Medical Research Ethics Committee (MREC) Utrecht by the Division of Pediatrics, UMC Utrecht. The need for informed consent was waived by the Division of Pediatrics, UMC Utrecht. The institutional MREC approved the study as non Medical Research Involving Human Subjects Act (WMO) (No. 20-031/C).

### Echocardiographic assessment

EchoPAC was used to screen echocardiographic reports and for echocardiographic reassessment. All echocardiographic measurements were performed according to recommendations from the American Society of Echocardiography. Echocardiographic measurements were performed on 2-dimensional parasternal long-axis views at the end of the diastole with closed mitral and aortic valve. Two-dimensional guided linear internal measurements of the IVS and LVPW were obtained perpendicular to the left ventricular long axis and measured at the level of the mitral valve leaflet tips. Z-scores corrected for body surface area were calculated automatically with a cardiac Z-score calculator, which has been validated in a large cohort of healthy infants^5,6,17,18^. Ultimately, CH was diagnosed if IVS and/or LVPW had a Z-score ≥ 2.0^4^. LV systolic function was assessed qualitatively on visual inspection “eyeballing” and/or quantitatively by fractional shortening (normal values between 28 and 46%) and/or ejection fraction (normal values between 56 and 78%) by an experienced pediatric cardiologist and was reported as normal or decreased. LV diastolic function was reevaluated in cases with thickening of both the IVS and LVPW using mitral inflow Doppler and Tissue Doppler Imaging (TDI) if this could be measured reliably. Diastolic dysfunction was characterized by an inversion of the mitral early and late diastolic E/A Doppler waves with a ratio < 0.8 or a mitral valve Doppler E/A ratio > 2. Tissue Doppler imaging signs of diastolic dysfunction were an IVS or LVPW E’ velocity < 8 cm/s^19^.

### Clinical data

Demographic and anthropometric characteristics, clinical signs and echocardiographic and prognostic data were collected from the patient files for each infant with CH. Etiologies of CH were subdivided into: maternal diabetes mellitus, malformation syndromes, metabolic disease, sarcomeric disease, congenital hyperinsulinism, neuromuscular disease and idiopathic CH. Each underlying cause was assessed for an association with hyperinsulinism and a subdivision was made in etiologies associated and not associated with hyperinsulinism^3^. For each infant with idiopathic CH it was examined whether genetic screening/diagnostics was performed including the outcome. Echocardiographic data included age of first echocardiography with CH, the distribution of left ventricular hypertrophy (Z-score ≥ 2 IVS and/or LVPW), the ratio between IVS and LVPW, asymmetric septal hypertrophy (ratio IVS:LVPW ≥ 1.5), decreased systolic and diastolic left ventricular function (LVF) and LVOTO^20^. Prognostic variables included were normalization of CH and mortality within the first year of life.

### Statistical analysis

Data were analyzed with IBM SPSS Statistics (v25.0, IBM Corp, Armonk, NY, USA). The Shapiro–Wilk test and Q–Q plots were used to test whether variables were normally distributed. Data were presented as median including percentiles 25–75, since almost all data were not normally distributed. Where appropriate, data were presented as number of patients with percentages. Differences between etiologies of CH associated and not associated with hyperinsulinism were analyzed by the Mann–Whitney *U* test (continuous data) and Fisher’s Exact Test (categorical data). One year survival of each etiology of CH was presented by a Kaplan–Meijer curve. Significance was determined as *P* value < 0.05.

## Results

### Baseline (Table 1)

**Table 1:** Baseline.

	n = 71
Demographics	
Male [*n* (%)]	44 (62)
Gestational age (weeks)	37.5 (34.9–39.7)*
Term 37–42 weeks [*n* (%)]	39 (61)
Moderate to late preterm 32–37 weeks [*n* (%)]	16 (25)
Very preterm 28–32 weeks [*n* (%)]	7 (11)
Extremely preterm < 28 weeks [*n* (%)]	2 (3)
Anthropometrics	
Length (cm)	54 (50–62)
Weight (kg)	4.2 (3.2–6.0)
BSA (m^2^)	0.26 (0.22–0.33)
Echocardiography	
Age of first echocardiography with hypertrophy (days)	20 (2–129)
*Interventricular septum thickness*	
mm	7.0 (5.8–8.7)
*Z-score*	+ 2.7 (2.1–3.5)
*Left ventricular posterior wall thickness*	
mm	5.4 (4.2–6.6)
*Z-score*	+ 3.1 (2.1–3.7)
Distribution left ventricular hypertrophy	
Z-score ≥ 2.0 [*n* (%)]	
Interventricular septum	14 (20)
Left ventricular posterior wall	10 (14)
Interventricular septum and left ventricular posterior wall	47 (66)
Ratio interventricular septum : left ventricular posterior wall	1.3 (1.1–1.7)
Asymmetrical septal hypertrophy [*n* (%)]	27 (38)
Left ventricular outflow tract obstruction [*n* (%)]	12 (17)
Left ventricular function	
Decreased left ventricular systolic function[*n* (%)]	23 (32)
Decreased left ventricular diastolic function [*n* (%)]	
E/A ratio < 0.8 or > 2.0	18/29 (62)**
E′ velocity < 8.0 cm/s	10/13 (77)**
Symptomatic [*n* (%)]	32 (45)

Data are presented as median with quartiles 25–75. Where appropriate, values are presented as number of patients with percentages.

*7 missing; **Only in cases with thickening of both interventricular septum & left ventricular posterior wall if this could be measured reliably.

Seventy-one infants with CH were included. Indications for echocardiography were prenatally diagnosed CH (n = 6), heart murmur (n = 9), enlarged heart on chest X-ray (n = 9), clinical suspicion of congenital heart disease or CH (n = 27) and screening for (suspected) syndromic disorder (n = 7) and others/unknown (n = 13).

### Etiologies (Fig. 1; Table 2)

**Figure 1:**
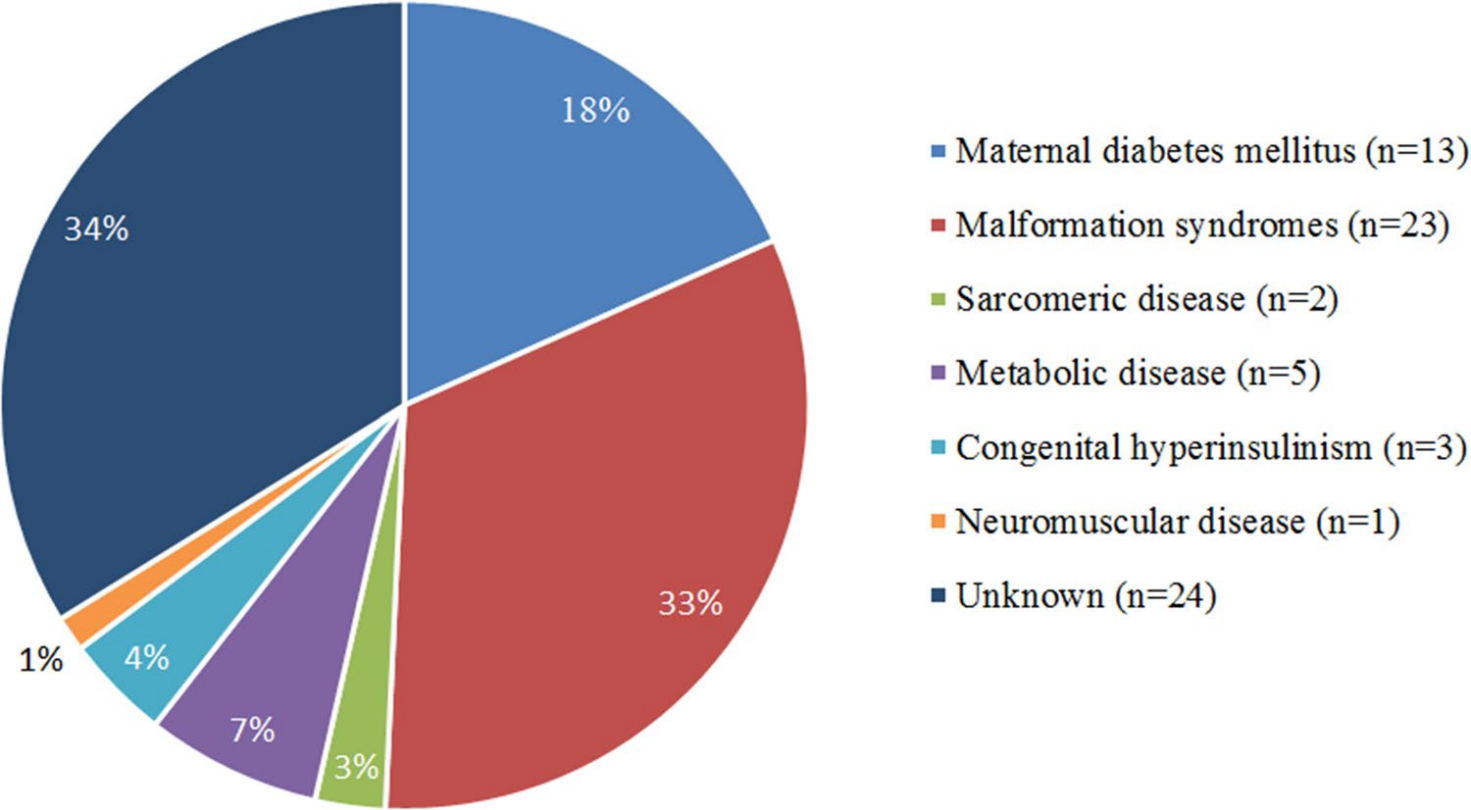
Underlying etiologies of cardiac hypertrophy in infants and their distribution. Malformation syndromes: Noonan (n = 12), Cantu (n = 3), Costello (n = 2), Berardinelli-Seip Congenital Lipodystrophy (n = 2), 1P36 chromosomal deletion (n = 1), Trisomie 18 (n = 1), Leprechaunism (n = 1), Beckwith-Wiedemann (n = 1); Sarcomeric disease: MYBPC3-gene mutations; Metabolic disease: Pompe (n = 3), GM1-gangliosidose (n = 1), VLCADD (n = 1); Congenital hyperinsulinism: Heterozygous mutation KCNJ11-gene (n = 1), hyperinsulinism (n = 1), after extreme dysmaturity (n = 1); Neuromuscular disease: Nemaline myopathy (mutation ACTA1-gene); Unknown: Variant of unknown significance (VUS) RYR2-gene, deletion RYR2-gene, paternal variant MYH7-gene (n = 2 siblings, 1 died), VUS MYH7-gene (n = 1).

**Table 2:** Etiologies of cardiac hypertrophy in infants.

	Maternal diabetes mellitus n = 13 (18%)	Malformation syndromes n = 23^1^ (32%)	Sarcomeric disease n = 2^2^ (3%)	Metabolic disease n = 5^3^ (7%)	Congenital hyperinsulinism n = 3^4^ (4%)	Neuromuscular disorder n = 1^5^ (1%)	Idiopathic n = 24^6^ (34%)
Echocardiography							
Age of first echocardiography with hypertrophy (days/months)	2d (1–3)	3.8m (2d-6.2m)	14d, 2.8m	6.7m (4.8–9.1)	2d, 3.4m, 3.5m	7.8m	20d (2d-2.2m)
*Interventricular septum thickness*							
mm	7.3 (6.1–8.1)	6.9 (5.8–8.7)	11.0, 16.0	11.0 (9.0–18.5)	11.6, 9.5, 6.4	5.4	6.4 (5.5–7.5)
*Z-score*	+ 3.0 (2.3–3.6)	+ 2.6 (2.0–3.5)	+ 5.1, + 6.4	+ 4.7 (3.5–6.9)	+ 5.1, + 4.0, + 2.7	+ 1.1	+ 2.3 (2.1–3.1)
*Left ventricular posterior wall thickness*							
mm	4.3 (3.4–5.2)	5.8 (4.8–6.5)	5.0, 6.6	12.5 (7.4–13.2)	8.0, 8.1, 3.6	6.4	5.2 (3.9–6.7)
*Z-score*	+ 2.1 (0.5–2.9)	+ 3.1 (2.3–3.4)	+ 2.8, + 3.6	+ 6.6 (3.7–7.0)	+ 4.9, + 4.7, + 1.3	+ 3.1	+ 3.1 (2.0–3.9)
Distribution left ventricular hypertrophy							
Z-score ≥ 2.0 [*n* (%)]							
Interventricular septum	6 (46)	2 (9)	0 (0)	0 (0)	1 (33)	0 (0)	5 (21)
Left ventricular posterior wall	2 (15)	3 (13)	0 (0)	0 (0)	0 (0)	1 (100)	4 (17)
Interventricular septum and left ventricular posterior wall	5 (39)	18 (78)	2 (100)	5 (100)	2 (67)	0 (0)	15 (63)
Ratio interventricular septum:left ventricular posterior wall	1.9 (1.3–2.5)	1.3 (1.1–1.5)	2.2, 2.4	1.1 (0.9–1.6)	1.5, 1.2, 1.8	0.8	1.2 (1.0–1.6)
Asymmetrical septal hypertrophy [*n* (%)]	9 (69)	6 (26)	2 (100)	2 (40)	2 (67)	0 (0)	6 (25)
Left ventricular outflow tract obstruction [*n* (%)]	0 (0)	8 (35)	1 (50)	0 (0)	1 (33)	0 (0)	2 (8)
Left ventricular function							
Decreased left ventricular systolic function [*n* (%)]	1 (8)	9 (39)	1 (50)	3 (60)	1 (33)	0 (0)	8 (33)
Symptomatic[*n* (%)]	6 (46)	9 (39)	2 (100)	4 (80)	0 (0)	0 (0)	11 (46)
Association with hyperinsulinism [*n* (%)]	13 (100)	6 (26)	0 (0)	0 (0)	3 (100)	0 (0)	Unknown
Prognosis							
Cardiac hypertrophy normalized [*n* (%)]	8 (89)*	9 (39)	0 (0)	0 (0)	2 (67)	1 (100)	18 (86)**
Age cardiac hypertrophy normalized (days/months/years)	3.3m (1.7–3.6)**	23.1m (17.3m-4.3y)	NA	NA	2.3y, 2.4y	1.8y	2.4m (1.7m-1.5y)**
Cardiac hypertrophy normalized in first year [*n *(%)]	8 (89)*	1 (4)	0 (0)	0 (0)	0 (0)	0 (0)	12 (57)**
All cause mortality [*n *(%)]	1 (8)	8 (35)	1 (50)	4 (80)	0 (0)	0 (0)	3 (13)^6^
Age of death (days/months)	5d	2.9m (2.1–4.4)	14d	8.8m (7.4–9.7)	NA	NA	2.3m (2.0–4.3)
Cardiac hypertrophy cause of death [*n *(%)]	0 (0)	4 (50)	1 (100)	3 (75)	NA	NA	1 (33)^6^

Data are presented as median with quartiles 25–75. Where appropriate, values are presented as number of patients with percentages.

^1^Noonan (n = 12), Cantu (n = 3), Costello (n = 2), Berardinelli-Seip Congenital Lipodystrophy (n = 2), 1P36 chromosomal deletion (n = 1), Trisomy 18 (n = 1), Leprechaunism (n = 1), Beckwith-Wiedemann (n = 1); ^2^ Mutations MYBPC3-gene; ^3^ Pompe (n = 3), GM1-gangliosidose (n = 1), VLCADD (n = 1); ^4^ Heterozygous mutation KCNJ11-gene (n = 1), CHARGE association (n = 1), after extreme dysmaturity (n = 1); ^5^ Nemaline myopathy (mutation ACTA1-gene); ^6^ Variant of unknown significance (VUS) RYR2-gene, deletion RYR2-gene, paternal variant MYH7-gene (n = 2 siblings, 1 died), VUS MYH7-gene (n = 1).

*4 missing; **3 missing.

An underlying cause was identified in two thirds of infants with CH. CH by *maternal diabetes mellitus* (n = 13) was diagnosed early after birth and mainly characterized by septal hypertrophy. In almost all infants with CH by maternal diabetes mellitus normalization occurred within the first months. The most common cause of CH in infants were *malformation syndromes* (n = 23). Both IVS and LVPW were hypertrophic in most infants with malformation syndromes. One third had LVOTO. The infant with Beckwith–Wiedemann was the only one in which CH normalized within the first year. In 2 infants CH was caused by *sarcomeric disease*. The IVS was extremely thickened and CH normalized in neither of the infants with sarcomeric disease. *Metabolic disease* was the cause of CH in 5 infants. CH was diagnosed relatively late. Both IVS and LVPW were extremely hypertrophic. Most infants were symptomatic. In none of the infants with metabolic disease CH normalized. Three girls had CH due to *congenital hyperinsulinism*. One infant had a heterozygous KCNJ11 mutation and insulin levels ranging from 71 to 152 mIU/L (reference value 5–25). Another infant with hyperinsulinism (insulin level 59 mIU/L) and CHARGE association showed decreased LVF and LVOTO at presentation. CH normalized with a betablocker. A third infant with hyperinsulinism after extreme dysmaturity needed high glucose intake, diazoxide and hydrochloorthiazide. One infant showed CH of the LVPW as a result of a *neuromuscular disorder*. In 24 infants the cause of CH remained *idiopathic*. Median gestational age at birth for infants with idiopathic cardiac hypertrophy was 37.0 weeks (IQR 25–75: 31.4–39.2). Ten infants with idiopathic cardiac hypertrophy were born preterm (47%, n = 3 gestational age unknown), including moderate to late (n = 4), very (n = 4) or extremely preterm (n = 2), respectively. The diagnosis was made relatively early after birth. IVS Z-scores were relatively low. In the majority CH normalized. In 5 infants with idiopathic CH genetic testing was performed. In 2 siblings a variant of unknown significance (VUS) and a deletion in the RYR2-gene and a paternal variant in the MYH7-gene was found. In 1 infant a VUS in the MYH7-gene was found. DNA-testing was normal for the other 2 infants.

### Etiologies of cardiac hypertrophy associated with hyperinsulinism (Fig. 2; Table 3)

**Figure 2:**
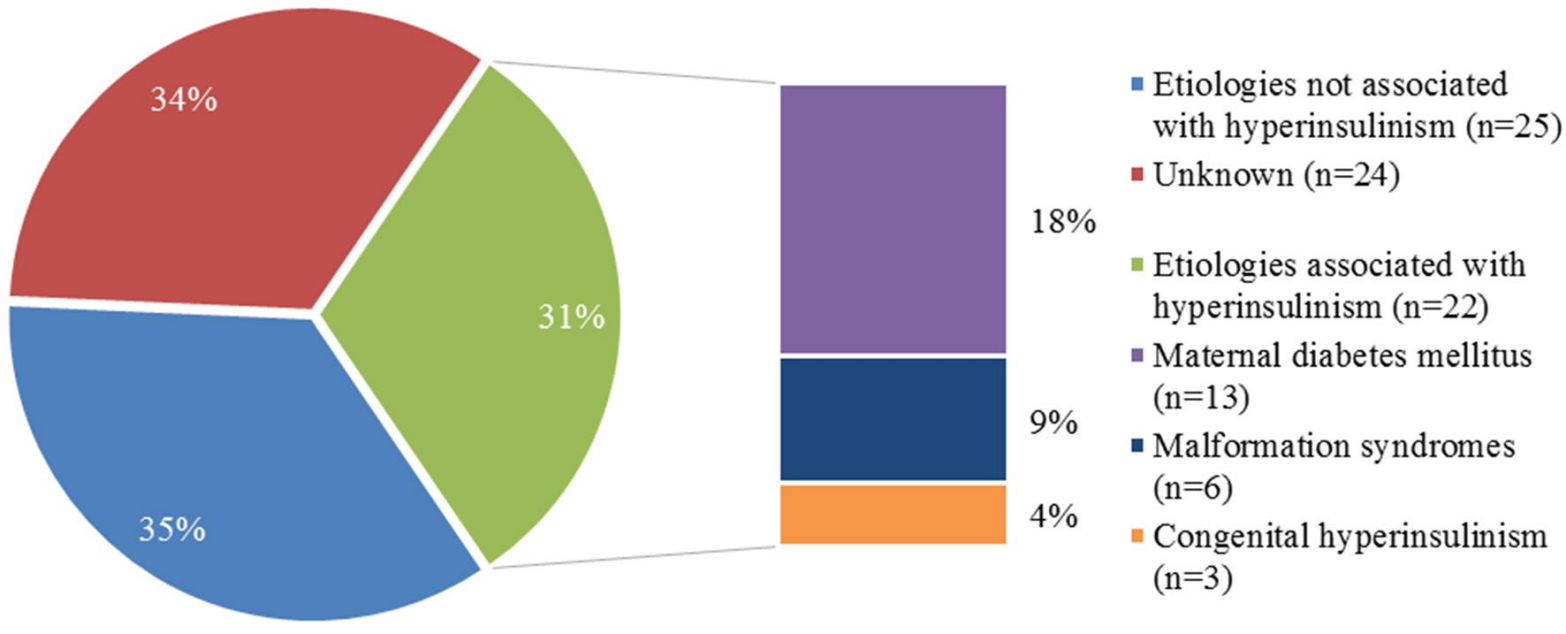
Underlying etiologies of cardiac hypertrophy in infants associated with hyperinsulinism. Malformation syndromes associated with hyperinsulinism: Costello (n = 2), Beckwith–Wiedeman (n = 1), Leprechaunism (n = 1), Berardinelli–Seip Congenital Lipodystrophy (n = 2).

**Table 3:** Differences between etiologies of cardiac hypertrophy associated versus not associated with hyperinsulinism.

	Etiologies of cardiac hypertrophy associated with hyperinsulinism (n = 22, 31%)*	Etiologies of cardiac hypertrophy not associated with hyperinsulinism (n = 25, 35%)	*p *value
Echocardiography			
Age of first echocardiography with hypertrophy (days/months)	2d (1d-1.8m)	4.4m (12d-7.7m)	< 0.01
*Interventricular septum thickness*			
mm	7.3 (6.2–8.1)	8.7 (6.1–10.9)	0.24
*Z-score*	+ 2.9 (2.4–3.6)	+ 3.4 (2.1–4.6)	0.45
*Left ventricular posterior wall thickness*			
mm	4.9 (3.6–5.5)	6.4 (5.4–8.1)	< 0.01
*Z-score*	+ 2.4 (1.0–3.0)	+ 3.3 (2.9–4.5)	< 0.01
Distribution left ventricular hypertrophy			
Z-score ≥ 2.0 [*n* (%)]			
Interventricular septum	8 (36)	1 (4)	0.01
Left ventricular posterior wall	3 (14)	3 (12)	1.00
Interventricular septum and left ventricular posterior wall	11 (50)	21 (84)	0.03
Ratio interventricular septum:left ventricular posterior wall	1.5 (1.3–2.0)	1.2 (1.1–1.5)	0.02
Asymmetrical septal hypertrophy [*n* (%)]	13 (59)	8 (32)	0.08
Left ventricular outflow tract obstruction [*n* (%)]	3 (14)	7 (28)	0.30
Left ventricular function			
Decreased left ventricular systolic function [*n* (%)]	5 (23)	10 (40)	0.23
Symptomatic [*n* (%)]	10 (46)	11 (44)	1.00
Etiology [*n* (%)]			
Maternal diabetes mellitus	13 (59)	0 (0)	
Malformation syndromes	6 (27)*	17 (68)	
Sarcomeric disease	0 (0)	2 (8)	
Metabolic disease	0 (0)	5 (20)	
Congenital hyperinsulinism	3 (14)	0 (0)	
Neuromuscular disease	0 (0)	1 (4)	
Prognosis			
Cardiac hypertrophy normalized [*n* (%)]	13 (59)	7 (28)	0.01
Age cardiac hypertrophy normalized (days/months/years)	102 (52–603)	904 (602–1830)	< 0.01
Cardiac hypertrophy normalized in first year [*n* (%)]	9 (41)	0 (0)	0.01
All cause mortality [*n* (%)]	3 (14)	11 (44)	0.03
Age of death (days/months)	65 (35–73)	138 (66–249)	0.09
Cardiac hypertrophy cause of death [*n* (%)]	1 (33)	7 (64)	0.54

Non-parametric data are presentated as median (with interquartile range 25–75%), group differences were tested by Mann–Whitney *U* test. Where appropriate, values are presented in number of patients with percentages, group differences were tested by Fisher’s Exact Test.

*Maternal diabetes mellitus (n = 13), malformation syndromes (n = 6, of which Costello n = 2; Beckwith–Wiedeman n = 1; Leprechaunism n = 1; Berardinelli–Seip Congenital Lipodystrophy n = 2) and congenital hyperinsulinism (n = 3).

In half of the identifiable causes of CH an association with hyperinsulinism was described including maternal diabetes mellitus (n = 13), malformation syndromes (Costello n = 2, Beckwith–Wiedeman n = 1, Leprechaunism n = 1, Berardinelli-Seip Congenital Lipodystrophy n = 2) and congenital hyperinsulinism (n = 3)^3^. Infants with an underlying cause of CH associated with hyperinsulinism were diagnosed at a younger age, showed more often sole hypertrophy of the IVS, had a less hypertrophic LVPW, and a higher IVS:LVPW ratio. In infants with an etiology of CH associated with hyperinsulinism CH normalized more often and faster.

### Prognosis (Fig. 3; Supplementary table 1)

**Figure 3:**
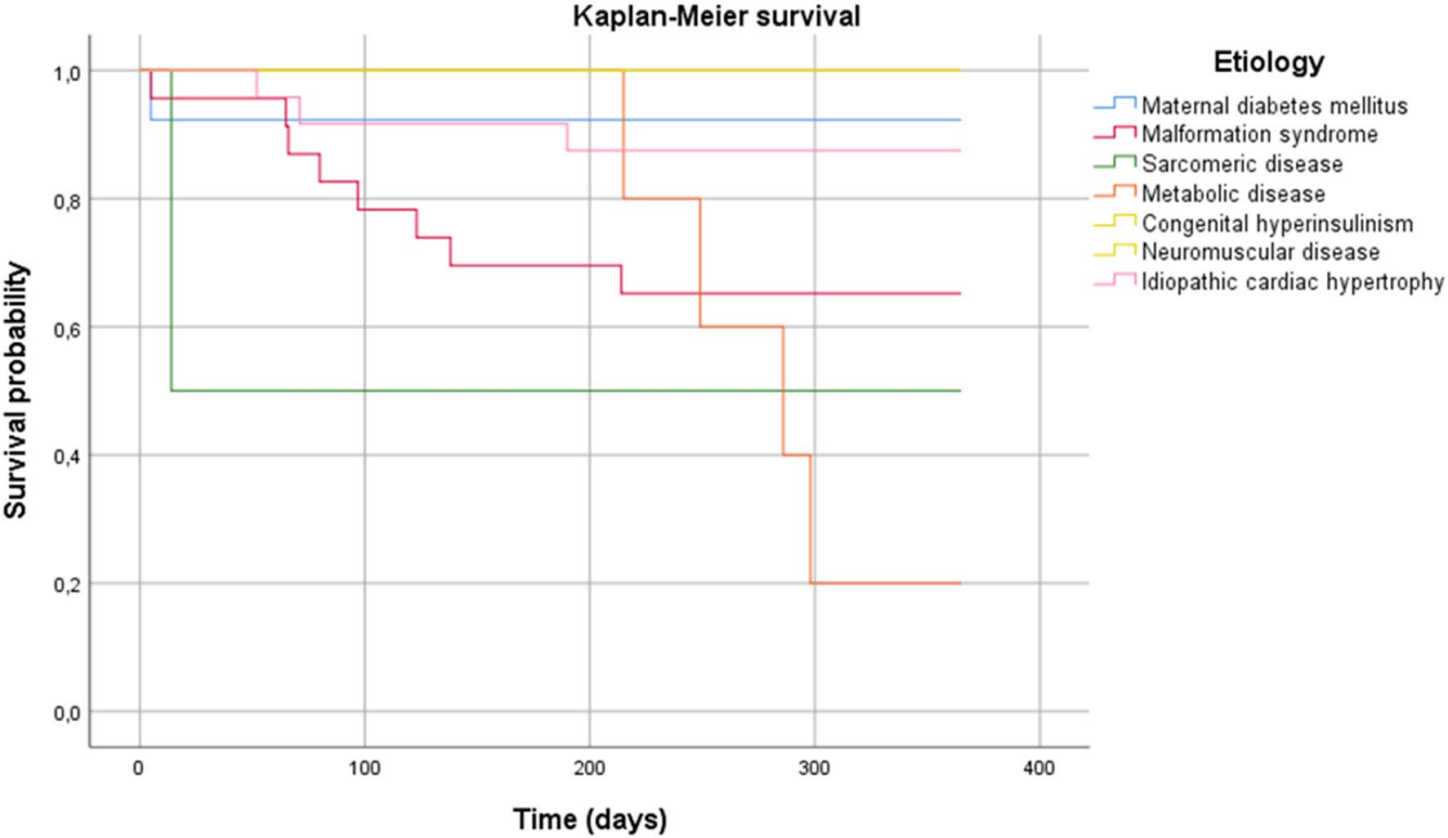
One-year survival of underlying causes of cardiac hypertrophy in infants.

Overall mortality rate in the first year was 24% (n = 17) and the median age of death was 3.2 months. Infants with malformation syndromes, sarcomeric and metabolic disease showed high all-cause mortality rates, whereas infants with CH by maternal diabetes mellitus, congenital hyperinsulinism, neuromuscular disorder and idiopathic CH had better survival chances. Nine infants (13%) developed severe progressive CH and died as a result of cardiac failure. CH as cause of death was based on clinical assumptions in 6 infants including Noonan (n = 2), Pompe (n = 2), VLCADD (n = 1) and Leprechaunism (n = 1), and on both clinical assumptions and the autopsy report in 3 infants including Noonan (n = 1), MYBPC3-gene sarcomeric disease (n = 1) and idiopathic CH (n = 1). Infants who died as a result of CH had a thicker IVS and LVPW and showed more often decreased left ventricular systolic function at initial presentation.

## Discussion

This study highlights the etiologies of CH in infants and their associations with hyperinsulinism, echocardiographic characteristics and prognosis. In this study, in the majority of infants with CH an underlying cause could be identified, including maternal diabetes mellitus, congenital hyperinsulinism, malformation syndromes, metabolic-, sarcomeric or neuromuscular disease. In half of these cases there was an association with hyperinsulinism^3^. Echocardiographic characteristics helped to differentiate between underlying causes of CH. Hyperinsulinism associated CH was diagnosed earlier and characterized by lower LVPW thickness, higher IVS:LVPW ratio and more often sole involvement of the IVS compared to CH not associated with hyperinsulinism. Normalization of CH and survival chances depended on the underlying cause and were more favorable in CH associated with hyperinsulinism compared to CH not related to hyperinsulinism. These findings confirm that establishing the correct diagnosis in infants with CH is extremely important since this has direct consequences for medical treatment and prognosis.

The heterogeneous etiology of CH in infancy is illustrated by the observation that in our cohort a total of 18 specific causes were identified in 71 infants. Although this sample size of infants with CH in our study was smaller in comparison to the Pediatric Cardiomyopathy Registry study of Colan et al. (n = 328), we focussed specifically on children below the age of 1 year and reported detailed results in terms of etiology specific echocardiographic findings and prognosis. In addition, in our study we identified underlying causes of CH in a larger proportion of our population when compared to the studies of Colan et al. and the population based longitudinal cohort study of Nugent et al. (66 vs. 31% and 58%, respectively)^11,12^. Compared to the study of Colan et al. we found a lower incidence of metabolic disease (7 vs. 15%), higher incidence of malformation syndromes (32 vs. 15%) and similar incidence of neuromuscular disease (1 vs. 1%). In both the study of Colan et al. and our study the causes that predominated in metabolic disease and malformation syndromes were Pompe disease and Noonan syndrome respectively. In our cohort only one infant with the neuromuscular disorder nemaline myopathy (mutation ACTA1-gene) was found, while Friedrich ataxia was the main cause of neuromuscular disease in the cohort with children of Colan et al.^11^ The relatively high incidences of metabolic disease and malformation syndromes and low incidence of neuromuscular disorders in our cohort of infants might be explained by the fact that metabolic or syndromic CH usually presents in infancy or early childhood, whereas those with neuromuscular disorders are more frequently diagnosed in adolescence^13^.

In one-third of our infants with CH the specific cause remained unknown. Over 50% of cases of apparently idiopathic CH in childhood are caused by mutations in cardiac sarcomere protein genes of which 17% of sarcomeric mutations were diagnosed before 1 year of age^13,21^. In contrast, in our cohort 2 infants (3%) were diagnosed with a MYBPC3-gene mutation. Variants and a deletion of unknown significance were found in 3 infants in the RYR2 and MYH7-gene. Importantly, only 5 infants with idiopathic CH (21%) underwent genetic testing. In the majority of idiopathic cases no indication for genetic testing existed, since CH normalized during follow-up. However, it may be possible that underestimated causes as sarcomeric and Danon disease (LAMP2 mutation) or currently undiscovered mutations could explain some of our idiopathic cases^13,15,21,22^. Analysis of a predefined panel of CH-related genes using next generation sequencing is generally recommended, including the most commonly involved genes in HCM (MYBPC3, MYH7 encoding sarcomeric proteins)^15,21,23^. In severe phenotypes of CH, more than one mutation can be found^24,25^. Interestingly, in a recent study by Aye et al., preterms showed a disproportionate CH during the first 3 months after birth, and greater changes in ventricular mass were associated with lower gestational age. This might explain CH in 10 of the idiopathic cases born prematurely^26^. In addition, the association of transient CH and corticosteroid treatment was recently described in a case report, and might possibly explain one of our currently idiopathic cases^27^.

Our study is the first to describe clear differences in echocardiographic findings of CH between infants with and without hyperinsulinemic disease. This attracted our interest, since we recently published a literature review in which we reported that CH is found in a broad spectrum of hyperinsulinemic diseases, including maternal diabetes mellitus, malformation syndromes with insulin resistence (of which Costello, Beckwith–Wiedemann, Leprechaunism, congenital generalized lipodystrophy) and congenital hyperinsulinism^3^. Previous studies including our literature review did not yet report echocardiographic findings and their differences between underlying causes of CH. Distinghuishing between etiologies associated and not associated with hyperinsulinism is of importance because of differences in outcome and therapeutic consequences, as CH by hyperinsulinism can respond favorably to insulin lowering therapies^3^.

Both Colan et al. and Lipshultz et al. studied the outcomes of CH in young childhood based on Pediatric Cardiomyopathy Registry data. Infants had significantly poorer survival (death/heart transplantation 19% at 1 year) than children presenting with CH after the age of 1 year^10,11^. This corresponds with the outcomes of our study were 24% of the infants died and all within the first year of life. The findings that infants with CH due to metabolic disease (death/heart transplantation 48% at 1 year) and malformation syndromes (19%) had worse survival rates than children with neuromuscular disorders or idiopathic CH were further substantiated in our study^10,11^. However, mortality rates in metabolic disease (80 vs. 48%) and malformation syndromes were higher (35 vs. 19%) in our study compared to death/heart transplantation rates in the study of Lipshultz et al., which might be explained by more serious cases in our cohort. In the longitudinal cohort study of Nugent et al. survival or no need for transplantation was 83% and 76% at 5 and 10 years after presentation with CH respectively^12^.

## Conclusions

We showed that underlying etiologies of CH can be identified in the majority of infants and include malformation syndromes, maternal diabetes mellitus, congenital hyperinsulinism, metabolic-, sarcomeric and neuromuscular disease. Noteworthy, underlying causes of CH were frequently associated with a broad range of hyperinsulinemic diseases and therefore hyperinsulinism should be included in the differential diagnosis of CH. Age at diagnosis and echocardiographic characteristics can help to distinghuish between the various underlying causes of CH associated and not associated with hyperinsulinism. Prognosis depends on the underlying cause of CH and is more favorable in infants with CH associated with hyperinsulinism. The results of this study are of added value in making the correct underlying diagnosis of CH in infants. This is of importance for prognosis and corresponding medical policy.

## Supplementary Information

Supplementary Information.

## Abbreviations

CH: Hypertrophic cardiomyopathy/cardiac hypertrophy
CHD: Congenital heart disease
HCM: Hypertrophic cardiomyopathy
IVS: Diastolic interventricular septum
LV: Left ventricular
LVF: Left ventricular function
LVOTO: Left ventricular outflow tract obstruction
LVPW: Diastolic left ventricular posterior wall
