# Supplementary material for: The etiology of cardiac hypertrophy in infants
Source: Sci Rep. 2021 May 19;11:10626. doi: 10.1038/s41598-021-90128-3 (PMC8134556; doi:10.1038/s41598-021-90128-3)
Supplement: Supplementary file 1 — Supplementary Information. [file 41598_2021_90128_MOESM1_ESM.docx]

**Supplementary table 1: Differences in cardiac morphology and function between infants who died and survived**

|  | **Infants who**  **died** | | | **Infants who**  **survived**  **(n=54, 76%)** | **P-value*** |
| --- | --- | --- | --- | --- | --- |
|  | **All-cause mortality**  **(n=17, 24%)** | **Cardiac hypertrophy cause of death**  **(n=9, 13%)** | |  |  |
| **Echocardiography**  Age of first echocardiography with hypertrophy, *days*  Interventricular septum thickness  *mm*  *Z-score*  Left ventricular posterior wall thickness  *mm*  *Z-score*  **Distribution left ventricular hypertrophy**  Z-score ≥ 2.0, *n (%)*  Interventricular septum  Left ventricular posterior wall  Interventricular septum & left ventricular posterior wall  Ratio interventricular septum:left ventricular posterior wall  Asymmetrical septal hypertrophy, *n (%)*  Left ventricular outflow tract obstruction, *n (%)*  **Left ventricular function**  Decreased left ventricular systolic function, *n (%)* | 39 (2-196)  7.8 (6.2-10.1)  +3.4 (2.4-4.5)  6.0 (4.5-8.1)  +3.2 (2.6-4.9)  3 (18)  0 (0)  14 (82)  1.4 (1.1-1.7)  7 (41)  5 (30)  12 (71) | 52 (8-203)  9.2 (7.4-14.8)  +3.8 (3.3-6.0)  6.7 (5.1-10.6)  +4.3 (3.0-6.0)  1 (11)  0 (0)  8 (89)  2.0 (1.0-2.0)  4 (44)  3 (33)  7 (78) | 20 (2-120)  6.9 (5.7-8.1)  +2.6 (2.1-3.5)  4.3 (4.2-6.5)  +3.0 (2.0-3.5)  11 (20)  10 (19)  33 (61)  1.2 (1.1-1.7)  20 (37)  7 (13)  11 (20) | | 0.72/**<0.01**  0.12/**<0.01**  0.07/**<0.02**  0.32/0.08  0.19/**0.05**  1.00/1.00  0.10/0.33  0.15/0.14  0.52/0.32  0.78/0.72  0.14/0.15  **<0.01/<0.01** |

*Non-parametric data are presented as median (with interquartile range 25-75%), group differences were tested by Mann-Whitney U test. Where appropriate, values are presented in number of patients with percentages, group differences were tested by Fisher’s Exact Test.*

* The first P-values (on the left side in the column) are the result of the comparison infants who died all-cause mortality and infants who survived. The second P-values (on the right side of the column) are the result of the comparison infants who died cardiac hypertrophy cause of death and infants who survived.
